# On the Use of Chloral Hydrate

**Published:** 1875-11

**Authors:** 


ON THE USE OF CHLORAL HYDRATE.—Dr. G. Leonardi (*Liguria Med.*) observes that, notwithstanding the value of the remedy, it cannot be denied that it is often misapplied. In London 4,400 pounds are consumed annually. The insufficient results hitherto observed are not attributable to the remedy, but to the carelessness of physicians, who, without properly understanding its physiological, therapeutical, and toxicological effects, administer it without distinction in the most different diseases. As yet the indications and contraindications for its employment have not been clearly set forth. Chloral hydrate acts, first, as an excitant; secondly, as an anæsthetic, especially on the cerebral centres. Its action, usually rapid, depends on its more or less good quality and on the varying individualities. It can be given either by the mouth or rectum; in the former case, mixed with syrup, in teaspoonful doses, or with water when given in the form of enema. The administration of chloral as an hypnotic is called for in all conditions which require the beneficial effects of recuperating sleep. It is contraindicated in cardiac debility with valvular lesions; in disorganizations in the mucous membrane of the digestive organs; in advanced diseases of the respiratory organs, etc. The medium dose varies between 30 and 75 grains. When given beyond 120 grains it acts like a deadly poison. It is an indispensable remedy in the hands of the intelligent physician, but may become a dangerous poison in those of the inexperienced, superficial practitioner.—*Med.-chir. Centralblatt.*—*New York Medical Journal.* E. F.

